# Invasive Hemodynamic Assessment of Cardiac Output State after MitraClip Therapy in Nonanaesthetized Patients with Functional Mitral Regurgitation

**DOI:** 10.1155/2016/6296972

**Published:** 2016-12-12

**Authors:** Frantisek Bednar, Tomas Budesinsky, Hana Linkova, Viktor Kocka

**Affiliations:** ^1^Cardiocenter, Third Faculty of Medicine, Charles University, Prague, Czech Republic; ^2^Department of Cardiology, University Hospital Kralovske Vinohrady, Prague, Czech Republic

## Abstract

*Background*. Surgical correction of mitral regurgitation (MR) can lead to postoperative low cardiac output state. We aimed to assess the acute hemodynamic changes after percutaneous MitraClip therapy (a unique model without influence of factors linked to surgical procedure) in patients with functional MR without the influence of general anaesthesia.* Methods*. We studied invasive hemodynamic parameters in 23 patients before procedure (conscious, nonsedated patients), during procedure (intubated patients), and the first day after MitraClip implantation (conscious, extubated patients).* Results*. Mitral valve clipping significantly increased cardiac index (CI) (from 2.0 ± 0.5 to 3.3 ± 0.6 L/min/m^2^; *p* < 0.01). Conversely, there was significant reduction in the mean pulmonary capillary wedge pressure (PCWP) (from 18.6 ± 5.7 to 10.5 ± 3.8 mmHg; *p* < 0.01), mean pulmonary artery pressure (from 29.8 ± 10.9 to 25.2 ± 10.3 mmHg; *p* = 0.03), and pulmonary vascular resistance index (from 531 ± 359 to 365 ± 193 dyn·s·cm^−5^/m^2^; *p* = 0.03).* Conclusions*. The functional MR therapy with percutaneous MitraClip device results in significant increase in CI (+66%) and concomitant decrease in PCWP (−42%). None of our patients developed low cardiac output state. Our results support the idea that significant part of low cardiac output state after cardiac surgery is due to surgery related factors rather than due to increase in afterload after MR elimination.

## 1. Introduction

Cardiac surgery for mitral regurgitation (MR) (either mitral valve repair or replacement) has a well described risk of deterioration of left ventricular (LV) function and acute postoperative low cardiac output (CO) state, especially in patients with preexisting LV dysfunction. The main known causes of low CO state are elimination of MR with resulting sudden increase in afterload but also several factors inherently linked to the injury due to cardiac surgery—depression of myocardial contractility after cardioplegic arrest, use of cardiopulmonary bypass, and interruption of annular-chordal-papillary muscle continuity. The principal mechanism leading to a decrease in LV systolic function after surgical correction of mitral regurgitation is considered to be the increase in afterload due to elimination of the low-impedance regurgitant flow into the left atrium [1–3].

Percutaneous mitral valve repair with MitraClip device provides a unique model to assess the acute hemodynamic changes solely attributable to elimination of mitral regurgitation. To date, we have limited data on acute hemodynamic changes after MitraClip measured by cardiac catheterisation methods. All previous published reports have significant limitations due to the fact that the measurements of CO before and after MitraClip implantation were performed under general anaesthesia and the studied cohorts were heterogeneous as it included patients with both organic and functional MR [4–6].

We therefore aimed to assess the acute hemodynamic effect after MitraClip therapy in patients with functional MR only and to assess invasively cardiac output state in a “real-world” situation without the influence of general anaesthesia.

## 2. Methods

Our study included 23 consecutive patients with functional MR indicated for MitraClip therapy according to current guidelines; all patients were discussed at the Heart Team meeting. In brief, patients were selected for percutaneous mitral repair if they had moderate to severe (3+) or severe (4+) MR and were symptomatic despite optimal medical therapy, including cardiac resynchronization therapy (CRT) if indicated, were judged inoperable or at high surgical risk and they fulfilled standard echocardiographic morphology criteria predicting procedural feasibility [7]. All patients have agreed to participate in this study and have signed informed consent; study was conducted in accordance with the Helsinki protocol.

### 2.1. MitraClip Procedure

The procedure was performed under general anaesthesia using transoesophageal echocardiography guidance and fluoroscopy. The MitraClip device (Abbott, Menlo Park, CA, USA) was advanced following an echo-guided transseptal approach to the left atrium and across the mitral valve to the left ventricle. The leaflets were grasped by pullback and arms of the clip were closed. The reduction of MR severity was assessed by Doppler echocardiography. A second clip was placed if further reduction of MR was needed. Acute procedural success was defined as successful clip implantation with MR reduction to grade 2+ or less by echocardiography.

### 2.2. Invasive Hemodynamic Measurements

The hemodynamic measurements were performed in 4 exactly defined time points:

- T1: a conscious nonanaesthetized patient immediately before the operation
- T2: 15 min after induction into the general anaesthesia
- T3: after clipping at the end of procedure still under GA
- T4: day 1 after procedure in fully conscious patient

Hemodynamic parameters were obtained using an arterial catheter (most often via radial artery), a balloon-tipped Swan-Ganz pulmonary artery catheter, and central venous catheter. Pulmonary capillary wedge pressure (PCWP) was assessed at end expiration and transducers were balanced by determining zero level at the midaxillary line. The Vigilance II Monitor (Edwards Lifesciences, USA) was used to calculate cardiac output using thermodilution principle and an average of six measurements was used. Systemic (SVRI) and pulmonary (PVRI) vascular resistance indexes and left (LVSWI) and right (RVSWI) ventricular stroke work indexes were calculated according to standard equations.

### 2.3. Follow-Up

Clinical follow-up including assessment of New York Heart Association (NYHA) functional status and echocardiography was obtained at 1 month after procedure in all included patients.

### 2.4. Statistical Analysis

Standard descriptive statistics were applied in the analysis: absolute and relative frequencies for categorical variables and mean supplemented by standard deviation for continuous variables. Wilcoxon paired test and Wilcoxon signed-rank test were used for the evaluation of statistical significance of differences between hemodynamic measurements at different time points. Paired *t*-test was used for comparison of LV ejection fraction at baseline and 1-month follow-up. The level of statistical significance was set at *p* < 0.05. Statistical analysis was computed using software SPSS 23.0.0.0 (IBN Corp., Armonk, NY, USA).

## 3. Results

The study analysed hemodynamic data from 23 consecutive patients who underwent MitraClip procedure in our centre. Baseline characteristics are summarized in Table 1. The aetiology of MR was functional in all patients and more than two-thirds had ischemic heart disease. The majority of patients had severe left ventricular dysfunction, and half of them have already history of congestive heart failure.

Procedure-related and clinical follow-up data are presented in Table 2. Acute procedural success was achieved in 21 (91%) patients. None of the patients needed inotropic support or prolonged mechanical ventilation at day 1 and all patients were hemodynamically stable. There were no access-site complications.

### 3.1. Invasive Hemodynamic Data

**Figure 1:**
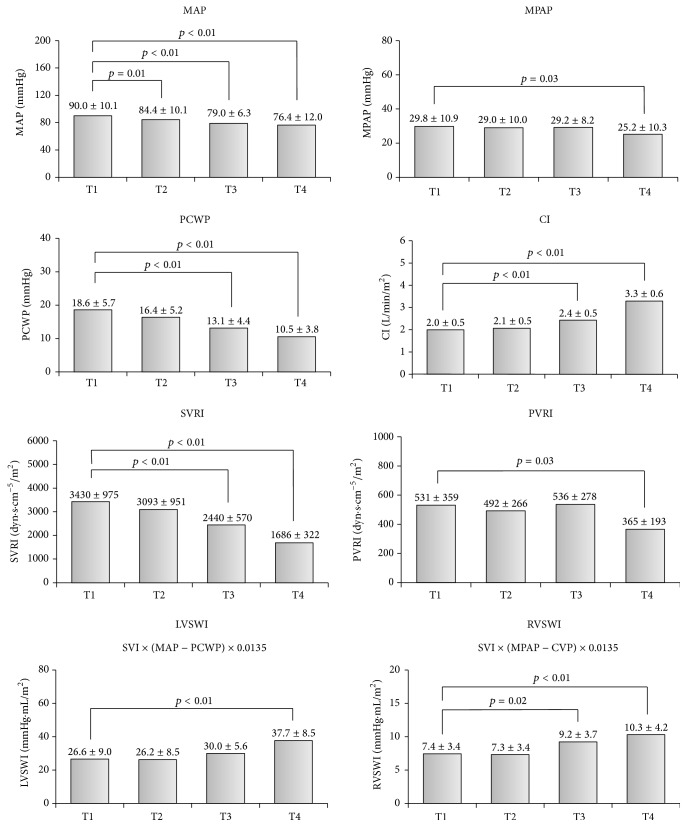
Invasive hemodynamic data and changes in the monitored time points. MAP, mean arterial pressure; MPAP, mean pulmonary artery pressure; PCWP, pulmonary capillary wedge pressure; CI, cardiac index; SVRI, systemic vascular resistance index; PVRI, pulmonary vascular resistance index; LVSWI, left ventricular stroke work index; RVSWI, right ventricular stroke work index; data are presented as mean ± SD.

**Figure 2:**
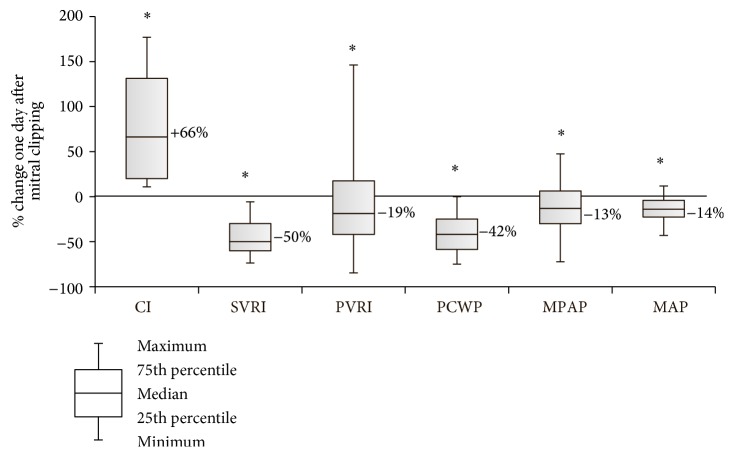
Percentage changes in hemodynamic parameters from baseline (T1) to day 1 after mitral clipping (T4). Data are presented as boxplots (median, interquartile range, minimum, and maximum). *∗* denotes a statistically significant difference (*p* < 0.05) by Wilcoxon signed-rank test.

Invasive hemodynamic data in the monitored time points are shown in Figure 1. Mitral valve clipping significantly increased cardiac index (CI) (from 2.0 ± 0.5 before procedure to 3.3 ± 0.6 L/min/m^2^ one day after procedure, *p* < 0.01). Similarly, MitraClip implantation significantly increased LVSWI (from 26.6 ± 9 to 37.7 ± 8.5, *p* < 0.01) and RVSWI (from 7.4 ± 3.4 to 10.3 ± 4.2 mmHg·mL/ m^2^, *p* < 0.01). Conversely, there was significant reduction in the mean PCWP (from 18.6 ± 5.7 to 10.5 ± 3.8 mmHg, *p* < 0.01), mean PAP (from 29.8 ± 10.9 to 25.2 ± 10.3 mmHg, *p* = 0.03), and mean arterial pressure (from 90 ± 10.1 to 76.4 ± 12 mmHg, *p* < 0.01) and simultaneously reduction in calculated SVRI (from 3430 ± 975 to 1686 ± 322 dyn·s·cm^−5^/m^2^, *p* < 0.01) and PVRI (from 531 ± 359 to 365 ± 193 dyn·s·cm^−5^/m^2^, *p* = 0.03). Percentage changes in hemodynamic parameters before procedure (T1) and next day after procedure (T4) are shown in Figure 2.

**Figure 3:**
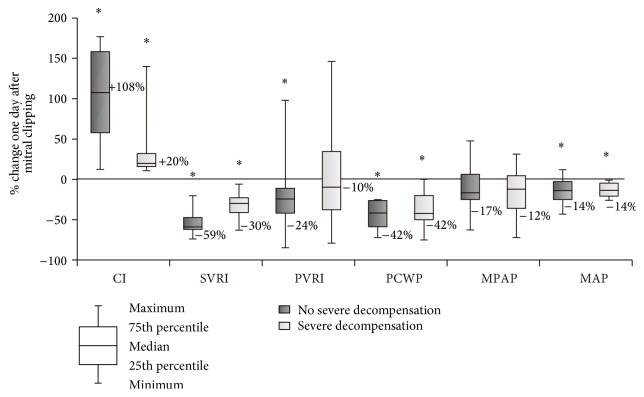
Percentage changes in hemodynamic parameters from baseline (T1) to day 1 (T4) between severe decompensated group of patients (defined as CI < 2.5 and PCWP > 15 mmHg before procedure) and the rest of the patients. Data are presented as boxplots (median, interquartile range, minimum, and maximum). *∗* denotes a statistically significant difference (*p* < 0.05) by Wilcoxon signed-rank test.

Figure 3 demonstrates percentage changes in hemodynamic parameters between severe decompensated group of patients (*n* = 13, defined as CI < 2.5 and PCWP > 15 mmHg before procedure) and the rest of the patients. The similar trend in the increase of CI and the decrease of PCWP, SVRI, PVRI, and mean pulmonary and systemic pressures was maintained also in the group of severe hemodynamically decompensated patients.

### 3.2. One-Month Follow-Up Data

**Figure 4:**
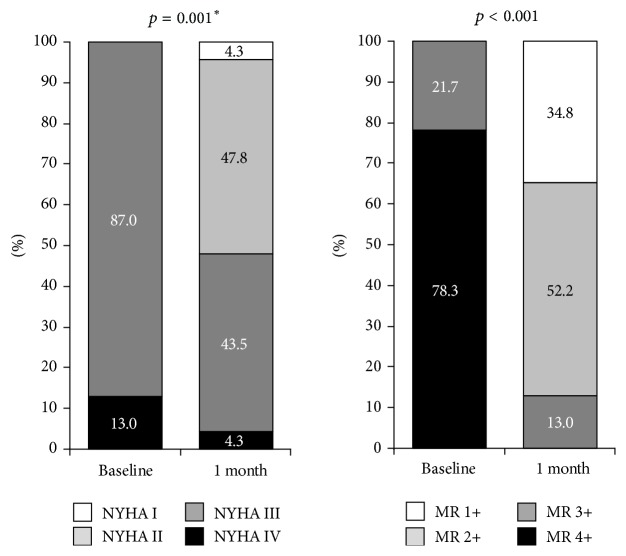
NYHA functional classification and MR severity at baseline and at 1 month. *∗* denotes Wilcoxon paired test.

Follow-up data were obtained in all included patients. Mitral regurgitation severity changes and improvement in NYHA classification are described in Figure 4. There was significant change in LV ejection fraction (from 34 ± 12% to 36 ± 11%, *p* = 0.018).

## 4. Discussion

This is the first study that reports acute hemodynamic changes after MitraClip therapy solely for functional mitral regurgitation without influence of general anaesthesia and measured invasively by right heart catheterisation. The principal findings are as follows: mitral clipping resulted in rapid increase of CI by 66% with concomitant reduction in SVRI by 50% and mean PCWP by 42% present already on first postoperative day. None of our patients experienced deterioration of haemodynamic stability despite the fact that most subjects had poor ventricular function (preprocedure LV ejection fraction was 34 ± 12%).

Our results are in agreement with previously published work by Siegel et al. [4] from the EVEREST trial, who described increased CI (from 2.6 ± 1.0 to 3.0 ± 1.0) and decreased SVR (from 1259 ± 531 to 1059 ± 479) after MitraClip implantation (*n* = 107). The principal difference is in patient population, especially with respect to MR aetiology (only 21% with functional MR and baseline LV ejection fraction was 59.8 ± 8.3%). Importantly, all hemodynamic data were obtained only under general anaesthesia with well-known effect on haemodynamic situation. Very similar results were reported by Gaemperli et al. [5], who described in their cohort of 50 patients increased CI by 32% and decreased PCWP by 20%. Also the limitations of this work are similar to the previous one; only 56% of patients had functional MR and baseline LV ejection fraction was high at 47 ± 18% and the last haemodynamic measurement after mitral valve clipping was obtained still under general anaesthesia.

To the best of our knowledge, our work is the first to invasively measure and describe the acute haemodynamic changes after MitraClip implantation in selected and homogenous cohort of patients with functional MR and severely depressed LV systolic function while carefully avoiding the confounding influence of general anaesthesia on haemodynamic situation. There is no deterioration including severe decompensated group of MR patients and in fact improvement in hemodynamic situation very quickly after this procedure. In summary, increase in forward cardiac output after MitraClip implantation indicates that the beneficial effect of end-diastolic unloading outweighs the negative effect of afterload increase. This is in contrast to well established belief (based on surgical experience) that elimination of MR leads to decrease of LV systolic function and low cardiac output state.

The important question remains: will the measured favourable hemodynamic parameters be translated to improved clinical outcomes? There is evidence that patients with increased CO and decreased filling pressures after MitraClip procedure have positive effect on LV remodelling with decrease in LV size and increase in LV ejection fraction [8]. These hemodynamic changes did result in improvement of clinical outcomes. The hemodynamic status is somewhat reflected by biochemical markers like natriuretic peptides and these levels correlate with clinical outcomes [9, 10]. The previous experience with MitraClip is encouraging; symptoms of heart failure (NYHA class reduction) and improvement of the life expectancy of high surgical risk patients compared with standard medical care were demonstrated [11–13].

This is in agreement with our experience; there was significant improvement in patient symptoms expressed by NYHA class after 1 month and significant improvement of LV ejection fraction.

## 5. Limitations

The main limitation of our work is the small number of studied subjects. This project was initiated only after our centre gained initial experience with this new method to avoid the influence of learning curve. We had carefully conducted all measurements but we are not able to account for possible changes in patient hydration. This is difficult as the patient is fasting prior to the procedure; the fluids administered during the procedure include usually poorly quantified catheter flushing and some patients do receive diuretic therapy after the procedure. Another technical issue might be linked to the use of thermodilution measurement; this may be affected by the presence of severe tricuspid regurgitation. However, only 4 of our patients had severe TR. In the postprocedure measurements, the usually present left-to-right shunting from iatrogenic atrial septal defect can underestimate CO.

## 6. Conclusions

The reduction of mitral regurgitation using percutaneous MitraClip device results in a quick improvement of cardiac output state in patients with functional mitral regurgitation. These results are independent of the influence of general anaesthesia. None of our patients developed low cardiac output state. Our results support the idea that significant part of low cardiac output state after cardiac surgery for mitral regurgitation is due to surgery related factors rather than being due to sudden increase in afterload. These findings may have important implications for the use of percutaneous MitraClip device in older patients with severe MR and poor LV function who have a high risk of developing postoperative low cardiac output state.

## Figures and Tables

**Table 1:** Baseline patients characteristics (*n* = 23).

Age, years	70 ± 6
Male (%)	70
Hypertension (%)	78
Coronary artery disease (%)	78
Atrial fibrillation (%)	74
History of HF (%)	48
LV ejection fraction (%)	34 ± 12
NYHA functional class (%)	
III	87
IV	13
MR aetiology, functional (%)	100

HF, heart failure; LV, left ventricular; NYHA, New York Heart Association; MR, mitral regurgitation.

**Table 2:** Procedure-related data and one-month follow-up data (*n* = 23).

APS (%)	91
Number of clips (%)	
1	78
2	22
Inotropic support (%)	0
In-hospital heart failure (%)	0
Early mitral valve surgery (%)	0
Hospital stay (days)	13 ± 9
In-hospital death (%)	0
	
*One-month follow-up data*	
LVEF (%)	36 ± 11
Rehospitalisation for HF (%)	0
One-month death (%)	0

APS, acute procedural success (defined as MR reduction after clip implantation to grade 2 or less); LVEF, left ventricular ejection fraction; HF, heart failure.
